# Continuous Heart Rate Monitoring for Automatic Detection of Life-Threatening Arrhythmias With Novel Bio-Sensing Technology

**DOI:** 10.3389/fcvm.2021.707621

**Published:** 2021-07-14

**Authors:** Ehud Chorin, Aviram Hochstadt, Arie Lorin Schwartz, Gil Matz, Sami Viskin, Raphael Rosso

**Affiliations:** ^1^Department of Cardiology, Tel Aviv Sourasky Medical Center and Sackler School of Medicine, Tel Aviv University, Tel Aviv, Israel; ^2^CardiacSense, Tel Aviv, Israel

**Keywords:** cardiac arrest, automatic arrhythmia detectors, photoplethysmography, ECG, sudden cardiac death

## Abstract

**Aims:** Assessing the effectiveness of novel bio-sensing technology (CardiacSense), for accuracy and reliability of automatic detection of life-threatening arrhythmias.

**Methods and Results:** This prospective study consisted of Eighteen patients (13 males and 5 females, mean age 59.4 ± 21.3 years) undergoing induction of ventricular tachycardia/fibrillation or provocation of transient ventricular asystole. We tested the detection of provoked ventricular arrhythmias by a wrist-worn watch-like device which uses photoplethysmography (PPG) technology to detect the cardiac rhythm. We used simultaneous electrocardiographic (ECG) recordings as gold standard for arrhythmia definition and confirmation of beat-to-beat detection. A total of 1,527 QRS complexes were recorded simultaneously by ECG and PPG. The overall correlation between the ECG (R-R intervals) and the PPG (G-G intervals) was high, with a correlation coefficient of R = 0.949 (*p* < 0.001). The device accurately detected all events of mimicked life endangering arrhythmias, including five events of transient (adenosine-induced) ventricular asystole as well as seven episodes of monomorphic ventricular tachycardia and 6 events of ventricular fibrillation.

**Conclusion:** This proof-of-concept study suggests that wearable devices using PPG technology, currently used to detect atrial fibrillation, may also have a role as automatic detectors of life-threatening arrhythmias.

## Introduction

Out of hospital sudden cardiac death (SCD) is very common worldwide and accounts for more than 5% of all crude mortality in the United States (1). Despite advances in the treatment and prevention of heart disease, the outcome of patients experiencing sudden cardiac arrest (SCA) remains poor, with rates of survival to hospital discharge ranging from 1.3 to 20.7% (2).

A major predictor of prognosis in SCA is response time, provision of cardiopulmonary resuscitation and a witnessed event (3). It is therefore imperative to shorten as much as possible the time to detection of SCA for an immediate initiation of CPR and preforming defibrillation as soon as possible (4). This need is emphasized in the American Heart Association cardiopulmonary resuscitation guidelines as the first link in the “out of hospital chain of survival” is “recognition of cardiac arrest and activation of the emergency response system” (4). Improving this link is crucial and most approaches are focused on public education of recognizing a cardiac arrest and initiating CPR (4). However, a vast percentage of SCA occurs during sleep or unwitnessed, thus making bystander early recognition impossible and significantly lowering chances of CPR success (5).

A continuous heart-rate monitoring device, comfortable enough to be worn all of the time and reliable enough to detect potentially life-threatening arrhythmias, could trigger the alarm that would start the chain of survival thus offering a better prognosis when a SCA occurs. We report here of our study with such a device, that uses photplatysmogrphy (PPG) technology, tested on patients with induced arrhythmias in a controlled setting as surrogate for life-threatening ventricular arrhythmias. Of note, heart-monitors using PPG technology are already in use for the detection of atrial fibrillation (6–11). However, to the best of our knowledge, this is the first time that the ability of a PPG-based “heart-watch” for detecting potentially life-threatening arrhythmias is reported.

## Methods

### Study Design and Patient Selection

This is a single-center, prospective study, assessing the effectiveness of novel bio-sensing technology (CardiacSense), for accuracy and reliability of automatic detection of life-threatening arrhythmias. The same PPG devices have been used to continuously detect sinus rhythm in ambulatory volunteers (7) and for the automatic detection of atrial fibrillation in patients at rest (6).

The study-group consisted of consecutive patients undergoing electrophysiological studies (EPS) or defibrillator implantation [with ventricular tachycardia/fibrillation (VT/VF) induction] or ablation procedures that included adenosine injection (provoking transient ventricular asystole). The study was approved by our Institutional Review Board (IRB number TLV 0066-16). All patients provided informed consent. Importantly, all the electrophysiologic studies performed during the course of the study, and all the attempts to provoke arrhythmias during the course of these procedures, were clinically indicated. For example, intravenous injection of high doses of adenosine (invariably provoking transient ventricular asystole due to sinus arrest or transient atrioventricular block) is standard practice during ablation of atrial fibrillation to test for pulmonary vein reconnection.

### The Bio-Sensing Technology (CardiacSense)

The CardiacSense is a wrist-worn watch-like device, specifically designed to detect cardiac arrhythmias. PPG is a simple optical technique that can be used to detect blood volume changes in the microvascular bed of tissues. Using this technology, it is possible to accurately detect the pulse rate and pulse pressure on a beat-by-beat basis. PPG is used for atrial fibrillation detection by other manufacturers, including the Apple Watch (8–11).

**Figure 1:**
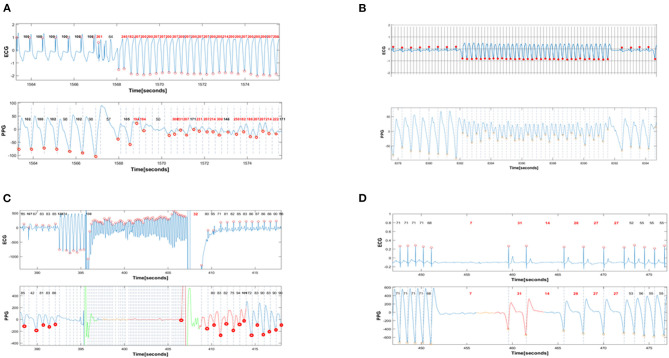
Simultaneous PPG and ECG recordings during provoked arrhythmias. In all panels, the red dots denote the timing of automatic detection of a QRS complex (R-R intervals) or a PPG signal (G-G intervals). The interval between red dots dictates the R-R (and G-G) interval that is automatically detected and annotated as beat-to-beat heart rate in beats/min. **(A)** shows the induction of monomorphic VT with programmed ventricular stimulation. The first 6 complexes are ventricular paced beats with a basic cycle length of 600 msec. All these beats are appropriately detected by PPG, with a heart rate of 100 beats/min in the ECG recorder and 98 −102 betas/min in the PPG recorder. Then there are 3 beats of ventricular extrastimulation with very short coupling interval. Only one of these 3 extrastimuli is detected by PPG. This is followed by an induced fast sustained VT (ventricular rate 210 beats/min). Except for the initial 9 beats of VT, almost all subsequent cardiac beats are also detected with a rate >200/min by PPG signals. **(B)**: Spontaneous ventricular tachycardia with a rate of 215 beats/min that terminates spontaneously after 10 s. All sinus complexes and all VT complexes detected by the ECG are also detected by PPG. Note the immediate decrement and subsequent alternans in PPG amplitude during VT, reflecting the reduced cardiac output during VT. Nevertheless, there is appropriate detection of VT by the PPG recorder. **(C)**: Induction of VF during ICD implantation. During sinus rhythm, eight beats of ventricular pacing are followed by a low energy shock delivered on the T-wave to induce VF that is eventually terminated by the ICD shock. The VF is falsely detected as “asystole” by PPG. **(D)**: Induced transient complete AV block provoked by adenosine and leading to 9 s of ventricular asystole. The prolonged ventricular asystole, as well as shorter subsequent pauses, are correctly detected by PPG.

The algorithm that detects a life-threating arrhythmia is based on two parameters: The first is the length of the RR interval (recorded as simultaneous interval between consecutive PPG signals, termed G-G interval), in order to detect episodes of predefined extreme bradycardia or tachycardia. The second, uses the signal to noise ratio of the PPG measurements, which correlates with the pulse pressure and thus correlate with cardiac output and tissue perfusion pressure. Prospective participants underwent simultaneous, continuous PPG (CardiacSense watch placed on the wrist) and ECG recordings during the entire procedure. The ECG recording was the gold standard used for analyzing the simultaneous PPG recording. Specifically, all cardiac intervals in ECG recordings (denoted as R-R interval) are compared with the quasi-simultaneous (delayed by a few milliseconds) PPG signals (denoted as G-G intervals) (Figure 1). Time to detection of cardiac arrest was set arbitrarily at 8 s to prevent false negative alert due to effect of rapid pacing or ventricular extrastimulation at the time of VT provocation during the EPS procedures.

#### Statistics

All data is summarized and displayed as number (and percentage) for categorical variables. Differences between RR and GG intervals was compared using the paired sample *t*-test. Accuracy was determined using the Pearson correlation test. Significant *p*-values were considered when *p* < 0.05. All calculations were done using SPSS v.24 from IBM, Armonk, Virginia.

## Results

### Study Population

Eighteen patients (13 males and 5 females, mean age 59.4 ± 21.3 years) participated in the study of simulated cardiac arrest events that were recorded with simultaneously with PPG and ECG (Table 1). The events simulating life-threatening arrhythmias included seven events of VT during electrophysiological studies, seven events of induced VF during defibrillator implantation and five events of transient ventricular asystole provocation adenosine injection.

**Table 1:** Baseline characteristics, *n* = 18.

**Variable**	
Age–mean (SD)	59.4 (21.3)
Male gender	13 (72.2)
DM	4 (22.2)
HTN	8 (44.4)
EF% mean (SD)	48.63 (12.71)
AF	6 (33.3)
Brugada syndrome	3 (16.6)
DFT after ICD implant	5 (27.7)

### Detection of Ventricular Tachycardia and Ventricular Fibrillation by the Device

During electrophysiological studies and defibrillation threshold testing (DFT), 7 VT events and 6 VF events were induced, all of which were detected by the PPG algorithm. Representative examples are shown in Figure 1. However, the VF events and one event of fast monomorphic (presumably hypotensive) VT, were detected as “asystole” rather than as very rapid tachyarrhythmias.

### Detection of Asystole by the Device

Adenosine test provoked 5 transient ventricular asystole events, all of which were detected by the PPG algorithm. Representative examples are shown in Figure 1D.

#### Measuring Accuracy of the PPG Detection or RR Interval Length

A total of 1,527 QRS complexes were recorded simultaneously by ECG and PPG during EPS, out of which 522 (34.2%) were recorded during procedures involving adenosine testing for provocation of asystole, 320 (20.1%) during procedures involving VF provocation and 685 (44.9%) during VT provocation. The overall correlation between the R-R and the G-G intervals was high, with a correlation coefficient of *R* = 0.949 (*p* < 0.001). There was a small but statistically significant difference between RR and GG intervals: the former being, as a group, shorter than the simultaneously recorded G-G intervals by 16.9 ± 209 msec, *p* = 0.002. Of note, these results do not apply to the VF and rapid VT episodes that appeared as “asystole” in the PPG recorder.

#### Measuring Accuracy of Cardiacsense Detection Algorithm

Out of the 18 events of cardiac arrest, all were detected by the PPG algorithm yielding a sensitivity of 100%. However, all VF episodes and one episode of rapid monomorphic VT were detected as “asystolic arrest” (rather than tachyarrhythmia-related arrest) due to absence of minimal amplitude PPG signals during tachyarrhythmias causing low- or no cardiac output.

## Discussion

This proof-of-concept study suggests that wearable devices using PPG technology, currently used to detect atrial fibrillation (6–11), may also have a role as automatic detectors of life-threatening arrhythmias. PPG-based heart-watches, like the Apple watch, have already been used to detect atrial fibrillation in large-scale studies (9, 11). We therefore speculated that the same devices could prove to be of use for the automatic detection of life-threatening ventricular arrhythmias.

Automatic arrhythmia detectors could draw the attention of household members (or individuals nearby in public places) with visual and audible alarms. The device could not only alert bystanders to an event of serious nature but could also instruct them to preform CPR, as this is often delayed even in populated settings. Furthermore, automatic arrhythmia detectors could communicate via mobile phone or Wi-Fi with Emergency Medical Services, providing them with PPG recordings of the event and with the patient's exact location. Automatic detectors of life-threatening arrhythmias could ultimately be designed to interact with drone networks delivering automatic external defibrillator (EAD) devices. A remaining concern remains the issue of false alarms.

The possibility of excessive false alarms must be addressed. With the newest generation of the Apple-Watch, which offers the possibility of ECG confirmation after automatic detection of atrial fibrillation by PPG sensors (also available in the device tested here), there were no events of false-positive detection of atrial fibrillation (9). To our knowledge, the Apple-Watch has not been used for detection of life-threatening arrhythmias. In a recent study of healthy volunteers who tested the present PPG-signal detector (using simultaneous Holter recordings as gold standard) while walking and/or performing daily activities, 0.7% of PPG recorded beats could not be matched to ECG re- corded beats and were considered false-detected (7). The issue of false alarms has been addressed by investigators of the wearable defibrillator, where a false alarm could actually trigger a painful inappropriate shock (12). When the wearable defibrillator senses a fast cardiac rhythm in the “VF zone,” it sounds an audible alarm. True VF is assumed to lead within seconds to loss unconsciousness. In contrast, patients with false-positive detection of VF can press a “shock-hold” button that will prevent the delivery of inappropriate shock for as long as the patient remains consiuous thus holding the button. Similar strategies for dealing with potential false alarm detections could be adopted for devices like CardiacSense. In the event of an automatic detection of a “life-threatening arrhythmia,” the patient would be prompted (by vibratory alarm) to perform an ECG via the same watch (as done in the Apple Watch). This ECG would prove or disprove the arrhythmia detection.

Our study has several limitations. The number of arrhythmic events tested was small. However, as a proof of concept, and knowing that each ECG signal triggers cardiac output that, in turn, triggers a PPG signal, it is fairly clear that additional arrhythmic events will add little information. A more important limitation relates to the fact that all patients were studied while resting, sedated, in a supine position. It remains to be demonstrated that arrhythmia detection is reliable in ambulatory and active patients. However, this particular PPG sensor has been shown to detect cardiac rhythm with fair accuracy in ambulatory patients in sinus rhythm (6). Finally, hypotensive VT and all VF events were misdiagnosed as “bradyasystolic arrest.” The last limitation is acceptable because the sequence of events that should follow any alarms triggered by “cardiac arrest detection,” regardless of the arrhythmia causing it, should ultimately lead to the deployment of AEDs designed to distinguish between shockable and non-shockable ventricular arrhythmias during cardiac arrest.

## Conclusion

The results of this proof-of-concept study suggest that PPG-based arrhythmia detectors, currently in use for the detection of atrial fibrillation, could be of use of the immediate detection of life-threatening arrhythmias.

## Data Availability Statement

The original contributions presented in the study are included in the article/supplementary material, further inquiries can be directed to the corresponding authors.

## Ethics Statement

The studies involving human participants were reviewed and approved by Tel Aviv Medical Center. The patients/participants provided their written informed consent to participate in this study.

## Author Contributions

EC, AH, AS, GM, SV, and RR have all participated in the data acquisition, study design, and writing of the research. All authors contributed to the article and approved the submitted version.

## Conflict of Interest

GM designed the detection algorithm for CardiacSense. SV is Chief Medical Officer for the cardiac arrhythmia section at CardiacSense. The remaining authors declare that the research was conducted in the absence of any commercial or financial relationships that could be construed as a potential conflict of interest.

## References

[1] Chugh SS Jui J Gunson K Stecker EC John BT Thompson B. Current burden of sudden cardiac death: multiple source surveillance versus retrospective death certificate-based review in a large U.S. Community. J Am Coll Cardiol. (2004) 44:1268–75. 10.1016/j.jacc.2004.06.029 15364331

[2] Nichol G Stiell IG Laupacis A Pham B Maio VJ Wells GA. A cumulative meta-analysis of the effectiveness of defibrillator-capable emergency medical services for victims of out-of-hospital cardiac arrest. Ann Emerg Med. (1999) 34:517–25. 10.1016/S0196-0644(99)80054-7 10499952

[3] Sasson C Rogers MA Dahl J Kellermann AL. Predictors of survival from out-of-hospital cardiac arrest: a systematic review and meta-analysis. Circ Cardiovasc Qual Outcomes. (2010) 3:63–81. 10.1161/CIRCOUTCOMES.109.889576 20123673

[4] Iwami T Nichol G Hiraide A Hayashi Y Nishiuchi T kajino K. Continuous improvements in “chain of survival” increased survival after out-of-hospital cardiac arrests: a large-scale population-based study. Circulation. (2009) 119:728–34. 10.1161/CIRCULATIONAHA.108.802058 19171854

[5] Kuisma M Jaara K. Unwitnessed out-of-hospital cardiac arrest: is resuscitation worthwhile? Ann Emerg Med. (1997) 30:69–75. 10.1016/S0196-0644(97)70114-8 9209229

[6] Hochstadt A Chorin E Viskin S Schwartz AL Lubman N Rosso R. Continuous heart rate monitoring for automatic detection of atrial fibrillation with novel bio-sensing technology. J Electrocardiol. (2019) 52:23–7. 10.1016/j.jelectrocard.2018.10.096 30476634

[7] Hochstadt A Havakuk O Chorin E Schwartz AL Merdler I Laufer M. Continuous heart rhythm monitoring using mobile photoplethysmography in ambulatory patients. J Electrocardiol. (2020) 60:138–41. 10.1016/j.jelectrocard.2020.04.017 32361522

[8] Marcus GM. The apple watch can detect atrial fibrillation: so what now? Nat Rev Cardiol. (2020) 17:135–6. 10.1038/s41569-019-0330-y 31873198

[9] Seshadri DR Bittel B Browsky D Houghtaling P Drummond CK Desai MY. Accuracy of apple watch for detection of atrial fibrillation. Circulation. (2020) 141:702–3. 10.1161/CIRCULATIONAHA.119.044126 32091929

[10] Wasserlauf J You C Patel R Valys A Albert D Passman R. Smartwatch performance for the detection and quantification of atrial fibrillation. Circ Arrhythm Electrophysiol. (2019) 12:e006834. 10.1161/CIRCEP.118.006834 31113234

[11] Perez MV Mahaffey KW Hedlin H Rumsfeld JS Garcia A Ferris T. Large-scale assessment of a smartwatch to identify atrial fibrillation. N Engl J Med. (2019) 381:1909–17. 10.1056/NEJMoa1901183 31722151 PMC8112605

[12] Adler A Halkin A Viskin S. Wearable cardioverter-defibrillators. Circulation. (2013) 127:854–60. 10.1161/CIRCULATIONAHA.112.146530 23429896

